# Overexpression of *FGFR2* contributes to inherent resistance to MET inhibitors in *MET*-amplified patient-derived gastric cancer xenografts

**DOI:** 10.3892/ol.2015.3601

**Published:** 2015-08-12

**Authors:** KAI LIU, XILIN SONG, MEIRONG ZHU, HENG MA

**Affiliations:** 1 Department of Gastrointestinal Surgery, Shandong Tumor Hospital, Jinan, Shandong 250117, P.R. China; 2 Intensive Care Unit, Jinan Central Hospital, Jinan, Shandong 250013, P.R. China

**Keywords:** MET, fibroblast growth factor receptor 2, gastric cancer, resistance

## Abstract

Gastric cancer is one of the most malignant diseases and one of the leading causes of cancer-associated mortality worldwide. Although advances have been made in surgical techniques, perioperative management and the combined use of surgery with chemotherapy and/or radiotherapy, patients with advanced stage gastric cancer continue to face poor outcomes. Furthermore, it was reported that *MET* gene amplification and overexpression predicted the sensitivity to MET inhibitors in gastric cancer. However, the identification of drug-resistant tumors has encouraged the pre-emptive elucidation of the possible mechanisms of clinical resistance. The current study assessed a number of patient-derived gastric cancer models with *MET* amplification and overexpression, including CNGAS028. The tumor tissues were subjected to microarray analysis (using single nucleotide polymorphism 6.0 and human genome U133 arrays) followed by western blotting. The results demonstrated that CNGAS028 xenograft tumors did not respond to treatment with a selective MET inhibitor. Additional analysis indicated that *FGFR2* overexpression contributed to the resistance to MET inhibitors. Furthermore, treatment with a combination of fibroblast growth factor receptor 2 and MET inhibitors inhibited the growth of CNGAS028 xenograft tumors *in vivo*. In conclusion, the current results aid in understanding the mechanism of inherent resistance to selective MET inhibitors as well as provide important information for patient selection and clinical treatment strategies.

## Introduction

Gastric cancer is the second most prevalent cause of cancer-associated mortality worldwide (1). However, over the past 50 years, the total incidence rates of gastric cancer have gradually decreased, particularly in developed countries. Furthermore, the disease most commonly occurs within the male population in developing countries, predominantly East Asia, South America and Eastern Europe (2). Conventional therapeutic strategies for gastric cancer include surgery, chemotherapy and radiation therapy (3). However, as gastric cancer has few symptoms during the early stages, the majority of patients are typically diagnosed once the cancer has progressed to an advanced stage. Despite undergoing surgical resection, tumors recur in a large number of patients, in such cases the median survival time following cytotoxic chemotherapy is <1 year. Therefore, the diagnosis and effective treatment of advanced gastric cancer continues to be a challenge for oncologists (4). Although the use of molecular targeted therapy has been studied in other common types of solid tumors, including non-small cell lung cancer and breast cancer, it has yet to be fully explored in gastric cancer (5).

*MET* was initially identified as an oncogene encoding the receptor tyrosine kinase (RTK) for hepatocyte growth factor. The *MET* gene has been identified on chromosome 7q21-q31, where it encodes a single precursor that is digested and glycosylated post-transcriptionally, resulting in an extracellular α-chain (50-kDa) linked to a transmembrane β-chain (140-kDa) via disulfide bonds. Oncogenic activation of *MET* suppresses apoptosis and promotes cell survival, proliferation, migration and differentiation, as well as gene transcription and angiogenesis (6,7).

Gain-of-function mutations in *MET* are uncommon in gastric cancer (8), with MET activation predominantly attributed to gene amplification (9). A previous used fluorescence *in situ* hybridization analysis in order to detect *MET* amplification, which was reported to occur in ≤4% of patients with gastric cancer (10). Various MET inhibitors have been investigated in clinical trials, which showed promising initial results indicating that MET may be a potential therapeutic target for the treatment of gastric cancer (11,12). An increasing number of pharmaceutical companies are focusing on the identification of novel small molecular c-MET inhibitors, including PF2341066 (Pfizer Ltd., Surrey, UK) and ARQ197 (ArQule Inc., Woburn, MA, USA) (13,14). However, the identification of drug-resistant tumors has encouraged the pre-emptive elucidation of potential mechanisms of clinical resistance. The present study describes a patient-derived gastric cancer model resistant to a selective MET inhibitor and attempts to determine the underlying mechanism.

## Materials and methods

### 

#### Establishment of patient-derived gastric cancer xenograft models

Female athymic BALB/c nude mice (n=200), aged 6–7 weeks, were purchased from Shanghai Laboratory Animal Centre Co., Ltd. (Shanghai, China). Mice were maintained under super-specific pathogen-free conditions and housed in barrier facilities on a 12 h light/dark cycle, with food and water provided *ad libitum*. All animal experiments were performed in accordance with protocols approved by the Shandong Tumor Hospital Experimental Animal Care and Use Committee. Fresh human gastric tumor specimens obtained from 83 Chinese patients that had undergone surgery were received from Shandong Tumor Hospital (Jinan, China) by Shandong Tumor Hospital Experimental Animal Center (Shandong, China) within 1 h of removal from the patients. The samples were cut into 3×3×3-mm sections, soaked in 50% Matrigel™ (BD Biosciences, Franklin Lake, NJ, USA) and subcutaneously implanted into the flank of the nude mice. The tumors were passaged when the tumor volume reached ~300 mm^3^. Tumor volumes were calculated using the following standard formula: Tumor volume = (length × width^2^)/2. Written informed consent was obtained from all patients and the study was approved by the ethics committee of Shandong Tumor Hospital Experimental Animal Center.

#### Detection of gene copy number and expression by microarray in established gastric cancer xenograft models

The GeneChip® genome-wide human single nucleotide polymorphism (SNP) 6.0 and human genome U133 plus 2.0 arrays (Affymetrix, Inc., Santa Clara, CA, USA) were used to analyze the genomic gene copy number and gene expression levels in all established patient-derived gastric xenograft tumors, respectively. MAS5 software (Affymetrix, Inc.) was used to analyze the U133 results. Gene profiling comparison was performed by calculating the fold change of the copy number and gene expression between these tumors. The data were processed using the aroma.affymetrix R package (version 2.13.0; http:www.aroma-project.org/), according to the methods described by Bengtsson *et al* (15).

#### Efficacy studies in gastric cancer xenograft models with MET amplification and overexpression

Gastric tumors (2-cm diameter) were aseptically resected from established patient-derived gastric cancer xenografts with *MET* amplification and overexpression, then minced into 3×3×3 mm pieces. Host mice were then anesthetized with isoflurane and a section of tumor was implanted into the left flank of each mouse. Each gastric model that developed tumors reaching 150–200 mm^3^ in size were randomized into the following four treatment groups (10 mice per group): Group 1, once-daily dose with vehicle by intravenous (i.v.) tail injection; and groups 2, 3 and 4, once-daily dose with 10, 20 and 30 mg/kg PHA665752 by i.v. tail injection, respectively. PHA665752, a selective MET inhibitor, was purchased from Selleck Chemicals (Houston, TX, USA). In a subsequent experiment, the CNGAS028 model was also treated with vehicle, 15 mg/kg PHA665752, the pan-fibroblast growth factor receptor (FGFR2) selective inhibitor NVP-BGJ398 (15 mg/kg once-daily, oral administration; Selleck Chemicals) or 30 mg/kg PHA665752 in combination with 15 mg/kg NVP-BGJ398, respectively. All treatments were continued for 21 days and the mice were sacrificed by CO_2_ inhalation 2 h after the last treatment.

#### Western blot analysis

The tumor tissues were resected 2 h following the final treatment with PHA665752 or/and NVP-BGJ398 on day 21 of the efficacy studies. The tumor tissues were then homogenized and lysed in cell lysis buffer (Bio-Rad Laboratories, Hercules, CA, USA) containing phosphatase inhibitor cocktail and proteinase inhibitor cocktail (Sigma-Aldrich, St. Louis, MO, USA), and the protein concentrations were determined using the bicinchoninic acid protein assay kit (Pierce Biotechnology, Inc. Rockford, IL, USA). Subsequently, equal quantities of protein (30 µg) were separated by sodium dodecyl sulfate/polyacrylamide gel electrophoresis on 8% gels, blotted on polyvinylidene difluoride membranes (Invitrogen Life Technologies, Inc., Carlsbad, CA, USA), then probed with monoclonal phosphorylated (p)-MET (1:1,000; cat. no. 3126), polyclonal p-FGFR2 (1:1,000; cat no. af3285; R&D Systems, Inc., Minneapolis, MN, USA), monoclonal MET (1:1,000; cat. no. 4560) and monoclonal FGFR2 (1:1,000; cat. no. 11835) rabbit anti-human antibodies. Subsequently, the membranes were incubated with goat anti-rabbit horseradish peroxidase-conjugated secondary antibodies (1:1,000; cat. no. 7074) and detected by chemiluminescence. Gel Doc™ XR+ (Bio-Rad Laboratories, Inc., Hercules, CA, USA) was used to visualize the western blots. All antibodies were purchased from Cell Signaling Technology, Inc. (Danvers, MA, USA), unless otherwise stated.

#### Statistical analysis

All data are presented as the mean ± standard deviation for the indicated number of independently performed experiments. Statistical analyses were conducted using GraphPad InStat software (version 5.0; GraphPad Software, Inc., San Diego, CA, USA). Student's t tests were performed and P<0.05 was considered to indicate a statistically significant difference.

## Results

### 

#### MET gene amplification and expression in Chinese patient-derived gastric cancer models

**Figure 1.**
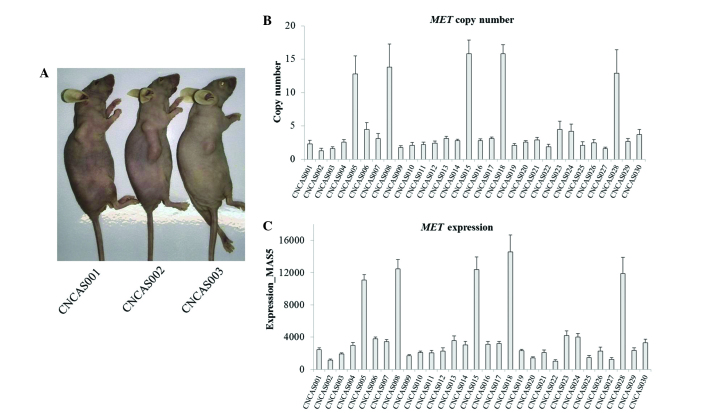
*MET* gene amplification and expression in 30 patient-derived gastric cancer models. (A) Images of the CNGAS001, CNGAS002 and CNGAS003 gastric cancer models. Analysis of the established gastric cancer models using (B) single nucleotide polymorphism 6.0 and (C) U133 plus 2.0 gene chips.

Chinese patient-derived gastric cancer models (n=30) were established from 83 gastric cancer specimens. The established models were termed CNGAS001-030. The CNGAS001, CNGAS002 and CNGAS003 mouse models are indicated in Fig. 1A. Microarray data from the SNP 6.0 and U133 plus 2.0 gene chips were used to analyze the genomic gene copy number and gene expression levels of all established models, respectively. The microarray data demonstrated that *MET* was highly amplified and expressed in 16.7% (5/30) of the Chinese gastric cancer xenograft models (Fig. 1B and C). From the results, it was observed that *MET* amplification was positively correlated with *MET* overexpression in the xenograft models.

#### High amplification and overexpression of MET predicts response to PHA665752 in patient-derived gastric cancer models

**Figure 2.**
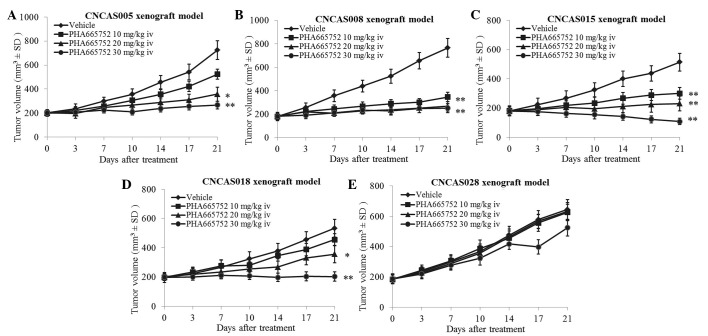
Tumor volumes of patient-derived gastric cancer models in response to PHA665752 or/and NVP-BGJ398 treatment. Nude mice bearing (A) CNCAS005, (B) CNCAS008, (C) CNCAS015, (D) CNCAS018 and (E) CNCAS028 tumors were treated with control vehicle or PHA665752 once-daily at the indicated doses by tail i.v. injection for 21 days. *P<0.05 and **P<0.01 vs. vehicle. i.v., intravenous; SD, standard deviation.

The present study analyzed the efficacy PHA665752 in the patient-derived gastric xenograft models with high *MET* amplification and overexpression (CNCAS005, CNCAS008, CNCAS015, CNCAS018 and CNCAS028). The results demonstrated that four gastric cancer xenograft models were significantly sensitive to PHA665752 treatment (P<0.05). However, the CNCAS028 model was resistant to PHA665752 (Fig. 2A–E; 30 mg/kg i.v. PHA665752 treatment group: P=0.008, P=0.006, P=0.004, P=0.007 and P=0.125, for the CNCAS005, 008, 015, 018 and CNCAS028 xenograft models, respectively).

#### High FGFR2 amplification and expression in the CNCAS028 model

**Figure 3.**
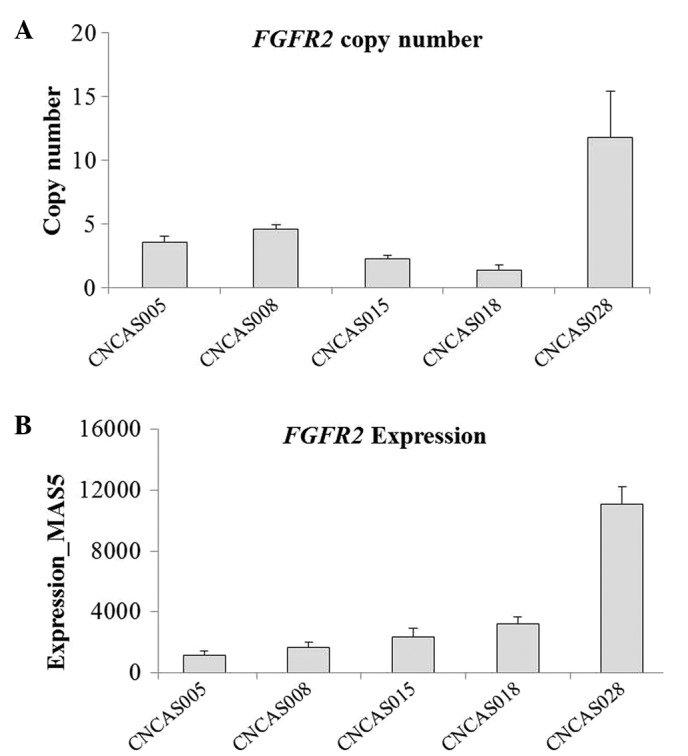
High *FGFR2* amplification and expression in CNCAS028 xenografts. CNCAS005, CNCAS008, CNCAS015, CNCAS018 and CNCAS028 tumors were resected from xenografts and then subjected to (A) single nucleotide polymorphism 6.0 and (B) U133 plus 2.0 gene chips. Mean ± standard deviation; n=5.

**Figure 4.**
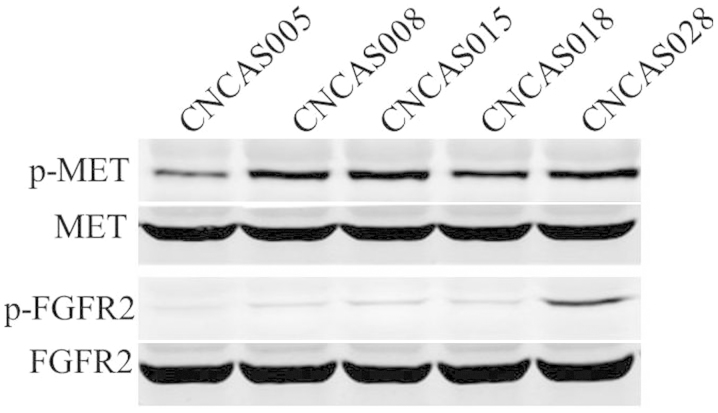
Expression level of p-MET, MET, p-FGFR2 and FGFR2 analyzed by western blotting in patient-derived gastric cancer models. (A) Expression levels of p-MET, MET, p-FGFR2 and FGFR2 in CNCAS005, CNCAS008, CNCAS015, CNCAS018 and CNCAS028 tumors. p-FGFR2, phosphorylated fibroblast growth factor receptor 2.

As indicated in Fig. 2A, the tumor growth of CNCAS028 was not inhibited by treatment with 30 mg/kg PHA665752 for 21 days. To explore the mechanism of the resistance to the selective MET inhibitor, the genome-wide gene profiles of CNCAS028 were compared with those of the other four gastric cancer models. It was identified that *FGFR2* was highly amplified and expressed in the CNCAS028 model, whereas *FGFR2* was expressed at a normal level and not amplified in the PHA665752-sensitive xenografts [normal *FGFR2* expression, copy number <5 and expression level <4,000 (as determined by GeneChip®); Fig. 3A and B]. These results were confirmed by western blot analysis (Fig. 4).

#### PHA665752 and NVP-BGJ398 combination treatment significantly inhibits tumor growth in the CNCAS028 model

**Figure 5.**
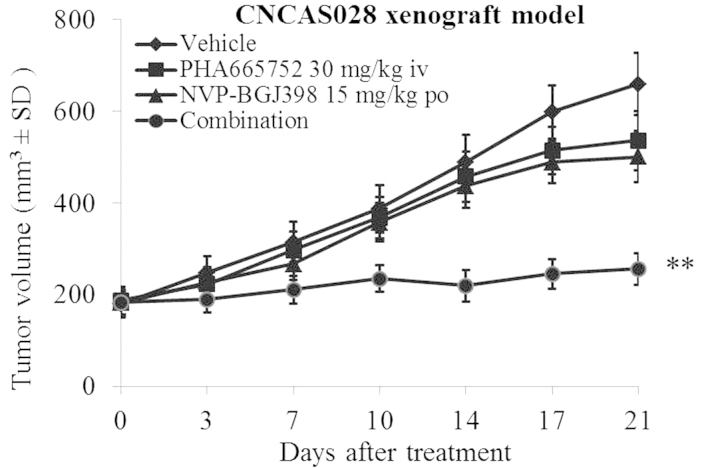
Tumor volumes of patient-derived CNCAS028 gastric cancer xenografts in response to PHA665752 or/and NVP-BGJ398 treatment, indicating the anti-tumor effect of each treatment. Nude mice bearing PHA665752-resistant CNCAS028 tumors were treated with 30 mg/kg PHA665752 once-daily by i.v. injection, 15 mg/kg NVP-BGJ398 orally or combination therapy for 3 weeks. **P<0.01 vs. vehicle. i.v., intravenous; SD, standard deviation.

To validate the association between *FGFR2* amplification and overexpression as well as PHA665752 resistance, PHA665752 was combined with a selective pan-FGFR2 kinase inhibitor (NVP-BGJ398) to treat the CNCAS028 model (16). As indicated in Fig. 5, treatment with 30 mg/kg PHA665752 was not able to inhibit tumor growth in the CNCAS028 model. Furthermore, treatment with 15 mg/kg NVP-BGJ398 only marginally inhibited tumor growth. By contrast, combined treatment with these two compounds significantly inhibited tumor growth following 21 days of treatment (P<0.01).

#### Effect of PHA665752 and/or NVP-BGJ398 on signaling transduction in patient-derived gastric cancer models

**Figure 6.**
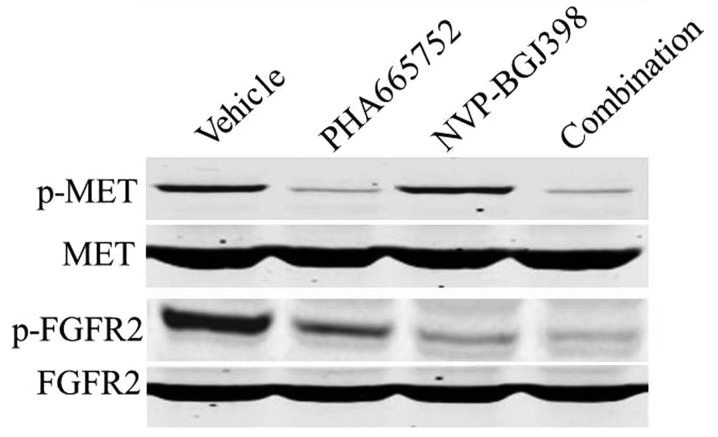
Expression level of p-MET, MET, p-FGFR2 and FGFR2 analyzed by western blotting in patient-derived gastric cancer models. Expression levels of p-MET, MET, p-FGFR2 and FGFR2 in CNCAS028 tumor tissues resected 2 h following the final treatment with PHA665752 and/or NVP-BGJ398 on day 21 of the efficacy study. p-FGFR2, phosphorylated fibroblast growth factor receptor 2.

To investigate the effect of PHA665752 and NVP-BGJ398 on downstream molecules of the phosphoinositide 3-kinase and RAS signaling pathways, western blot analysis was used to observe changes in the phosphorylation status and total protein expression levels of the tumor tissues. The results demonstrated that all five patient-derived gastric xenograft models highly expressed p-MET and the CNCAS028 xenograft also highly expressed p-FGFR2 (Fig. 4). Western blot analysis also identified that treatment with PHA665752 inhibited the phosphorylation of MET in all five gastric tumor models. In addition, the expression of p-FGFR2 was markedly inhibited by NVP-BGJ398 or combination treatment in the CNCAS028 model (Fig. 6).

## Discussion

Gastric and gastroesophageal cancer affect 1 million individuals worldwide every year and are the second most common cause of cancer-associated mortality (17). Targeted therapies have been developed and incorporated into the standard treatment strategies for other types of solid cancer, such as lung or breast. However, such therapies (including MET inhibitors) are only now being examined in the context of gastric and gastroesophageal cancer (18). The current investigations identified *MET* gene amplification in 5/30 (16.7%) patient-derived gastric cancer xenografts. These results indicated the therapeutic potential of MET inhibitors in gastric cancer. Additional analysis identified that a selective MET inhibitor (PHA665742) was able to significantly inhibit tumor growth in 4/5 gastric cancer models with *MET* amplification. These results are consistent with a previous study, which demonstrated that *MET* amplification was associated with the response of the MKN45 gastric cancer cell line to PHA665752 treatment (19).

Aberrant RTK expression produces growth and survival signals that are essential for the pathogenesis and progression of various types of cancer. Furthermore, cancer patients treated with targeted inhibitors of key oncogenic kinase drivers, including imatinib, gefitinib and erlotinib, have exhibited promising clinical outcomes (20). However, based on the precedence set by agents such as imatinib in chronic myeloid leukemia and erlotinib in lung adenocarcinoma, inherent resistance may potentially limit the application of single agent therapies (21). The elucidation of novel oncogenic drivers may have extensive implications for targeted therapy. Corso *et al* (22) reported that activation of HER family members in MET-addicted cancer cells, subsequent to MET inactivation, resulted in increased cell viability *in vitro* and recovered tumorigenicity *in vivo*. In addition, Lee *et al* (23) reported that a novel *SND1-BRAF* fusion gene exhibited resistance to the MET inhibitor PF-04217903 in GTL16 cells via RAS/RAF/ERK signaling pathway activation. By contrast, the current study determined that a *MET*-amplified CNCAS028 model was resistant to MET inhibitor PHA66575 as a result of *FGFR2* gene amplification and overexpression. Inhibition of FGFR2 signaling in this xenograft model recovered its sensitivity to PHA665752.

In conclusion, MET and FGFR2 coactivation may increase resistance to targeted therapy, possibly due to activation of multiple growth and survival signaling pathways. These findings indicate that combination therapy with MET and FGFR2 inhibitors may be a promising strategy to treat inherent resistance to MET inhibitors in cases of gastric cancer harboring *MET* and *FGFR2* amplification. Future studies should be performed to investigate whether similar results could be obtained in an acquired resistant model.
